# Examining multimorbidity contributors to dementia over time

**DOI:** 10.1002/alz.14589

**Published:** 2025-02-23

**Authors:** Matthias Klee, Sheila Markwardt, Miriam R. Elman, Ling Han, Anja K. Leist, Heather Allore, Ana Quiñones

**Affiliations:** ^1^ Department of Social Sciences University of Luxembourg Esch‐sur‐Alzette Luxembourg; ^2^ OHSU‐PSU School of Public Health Oregon Health & Science University Portland Oregon USA; ^3^ Department of Internal Medicine Yale School of Medicine New Haven Connecticut USA; ^4^ Department of Biostatistics Yale School of Public Health New Haven Connecticut USA; ^5^ Department of Family Medicine Oregon Health & Science University Portland Oregon USA

**Keywords:** ADRD, Alzheimer's disease and related dementias, longitudinal extension of the average attributable fraction ( LE‐AAF), multimorbidity, population attributable fraction ( PAF), population attributable risk

## Abstract

**INTRODUCTION:**

Multimorbidity is associated with increased risk of dementia, but previous estimation of the joint contribution of constituent conditions to dementia incidence did not model additive contributions or temporal proximity in the sequential onset of conditions.

**METHODS:**

Data were analyzed from 9944 Health and Retirement Study participants and Medicare fee‐for‐service beneficiaries, ages 68–99, without Alzheimer's disease and related dementias (ADRD) at baseline, from 1998–2016. ADRD and chronic condition were encoded using validated claims algorithms. We estimated the absolute contribution of eight conditions to ADRD with the longitudinal extension of the average attributable fraction (LE‐AAF).

**RESULTS:**

Hypertension, acute myocardial infarction, atrial fibrillation, diabetes, heart failure, ischemic heart disease, stroke, and arthritis additively accounted for 71.8% (95% confidence interval [CI]: 62.9%–79.1%) of ADRD incident cases based on LE‐AAF.

**DISCUSSION:**

Our findings suggest that multimorbidity plays a pivotal role in ADRD incidence. Targeting constituents of a cardiovascular path to dementia may contribute most to lowering dementia risk.

**Highlights:**

- Most dementia cases (71.8%) were attributable to eight chronic conditions.
- Hypertension was the largest contributor to dementia risk.
- Confidence intervals were smallest for constituents of a cardiovascular path to dementia.
- Longitudinal extension of the average attributable fractions (LE‐AAFs) explicitly consider longitudinal patterns of comorbidities.
- Acute myocardial infarction did not contribute significantly to dementia incidence.

## 1 BACKGROUND

Previous research suggests that globally, many people living with Alzheimer's disease and related dementias (ADRD) also live with chronic comorbid conditions such as hypertension, heart disease, or diabetes.^1^, ^2^ As an example, over one‐third of Medicare beneficiaries 65 and older with ADRD had comorbid diabetes (37%) or heart failure (34%).^3^ Some of these conditions are widely established risk factors for ADRD, with evidence of etiological linkages via inflammatory pathways or vascular damage, backed by the efficacy of antidiabetic or antihypertensive agents in reducing ADRD risk.^4^, ^5^, ^6^, ^7^ Moreover, chronic conditions manifest in varying chronological sequences and accumulate at different rates starting in a preclinical stage.^8^ Evidence of associations of chronic conditions with increased ADRD risk and contributions to dementia incidence have been reported previously for cardiometabolic multimorbidity (i.e., with diabetes, heart disease, stroke, or myocardial infarction), as well as atrial fibrillation, arthritis, heart failure, and hypertension.^1^, ^9^, ^10^, ^11^, ^12^, ^13^, ^14^, ^15^

The presence of cardiometabolic multimorbidity is generally associated with higher ADRD risk, which generalizes to most common dementia subtypes.^16^, ^17^ Still, it is currently unclear if increased ADRD risk in multimorbidity reflects accumulated inequality, severity of chronic conditions, or reverse causation at a given point in time.^18^ To gain a better understanding of dementia risk in late life, estimating the absolute contributions of chronic conditions, that is, the contribution irrespective of potentially comorbid risk factors over the observational period, requires longitudinal assessments of changing morbidity patterns over significant portions of the lifespan (i.e., mid‐ to later life). Eventually, quantifying the population level absolute contribution of individual or multiple co‐occurring chronic conditions to dementia incidence is crucial for guiding efficient resource allocation for dementia prevention efforts.^8^

Understanding precursors to incident ADRD requires estimating contribution of chronic conditions as risk factors of dementia as a clinical syndrome.^1^, ^19^, ^20^, ^21^, ^22^ Although vascular dementia (VaD; i.e., dementia characterized by clinically relevant impairment following cerebrovascular insult) is etiologically distinguished from Alzheimer's disease (AD) or other dementias, downstream contribution of chronic conditions to and interaction with hallmark indicators of AD including neurodegeneration, amyloid deposition, and hippocampus‐mediated cognitive impairment are increasingly recognized.^23^, ^24^, ^25^, ^26^, ^27^ Moreover, co‐pathologies, for example, occurrence of Lewy bodies in AD; shared risk factors, for example, VaD and AD; and involvement of inflammatory processes in AD‐related pathophysiology, reinforce the need to focus on incident ADRD.

**RESEARCH IN CONTEXT**

1. **Systematic review**: Evidence regarding the associations of chronic conditions with dementia risk was assessed in a literature search of PubMed with the terms risk factors, dementia, and population attributable fraction. If available, estimated total population attributable fractions were screened and compared with the present findings.
2. **Interpretation**: Our findings suggest that 71.8% of dementia cases in Medicare fee‐for‐service beneficiaries 68 years of age and older to be attributed to the cumulative burden of eight risk factors of dementia, when considering multiple temporal sequences of constituent chronic conditions. Hypertension was highly prevalent in the study population and contributed most to dementia incidence.
3. **Future directions**: With updated relevance of chronic conditions for dementia risk, continued and concerted efforts to effectively target and reduce risk factors in individuals and populations are warranted. Suggested preventive potential requires further assessment of efficacy of multimorbidity management for ADRD risk prevention.

To capture the joint contribution of chronic conditions to dementia as a clinical syndrome, frequently co‐occurring conditions characterized previously as risk factors—diabetes, ischemic heart disease, stroke or transient ischemic attack (STIA), acute myocardial infarction (AMI), atrial fibrillation, rheumatoid arthritis or osteoarthritis, heart failure, and hypertension— require concomitant inclusion to models.^1^, ^9^, ^10^, ^11^, ^12^, ^13^, ^14^, ^15^ The contributions of chronic conditions (multimorbidity) to ADRD have been estimated frequently by computing population attributable fractions (PAFs), which increase with the prevalence of chronic conditions and relative association strength of chronic conditions with ADRD.^28^, ^29^ Previous research suggests high PAFs of up to 20% for individual chronic conditions such as hypertension, and up to ≈40% for weighted combined contributions of chronic conditions and other risk factors.^30^, ^31^, ^32^, ^33^ Although reported PAFs sometimes adjust for concurrent factors, they do not account for the sequential accumulation of multimorbidity of chronic conditions and are not additive, with sums potentially exceeding 100%.^34^ Furthermore, commonly applied dimensionality reduction strategies to derive weights and adjust for the communality of individual PAFs when computing joint contributions of chronic condition combinations to ADRD risk imperfectly account for longitudinal coevolution of multimorbidity.^1^, ^2^, ^33^, ^35^ Similarly, nominal chronic condition groupings are agnostic to temporal ordering of chronic conditions and may be biased depending on a priori defined cutoffs or chronic condition combinations.^17^, ^36^, ^37^, ^38^, ^39^

Recent methods have been developed to overcome these issues, accounting for the sequence of occurrence of conditions over time with the longitudinal extension of the average attributable fraction (LE‐AAF).^40^, ^41^, ^42^ In the present study, we applied LE‐AAF to determine absolute estimates of the average PAF of eight chronic conditions regarding ADRD incidence in a nationally‐representative U.S.‐based sample. This allowed the examination of the additive contribution of known pathologic pathways in which chronic conditions may alter late‐life ADRD risk, and to differentiate individual contributions in the presence of multimorbidity.

## 2 METHODS

### 2.1 Study population

We used Medicare fee‐for‐service claims for beneficiaries who participated in the Health and Retirement Study (HRS) between 1998 and 2016. ADRD and chronic condition codes were based on validated standardized claims algorithms from the Chronic Conditions Data Warehouse (CCW), provided by the Centers for Medicare & Medicaid Services (Table S1). Since October 1, 2015, diagnostic codes are based on the International Classification of Diseases, 10th Revision, Clinical Modification (ICD‐10‐CM). Previous codes were based on ICD‐9‐CM.^43^ Resources can be found online.

**FIGURE 1:**
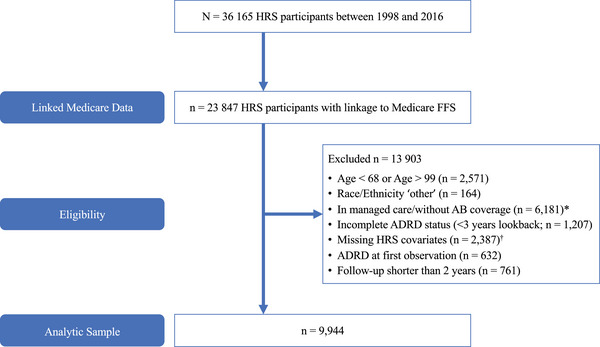
Flowchart of inclusion. *CCW algorithms cannot be applied to participants enrolled in managed care or without Medicare Parts A and B coverage, resulting in missing chronic condition status. ^†^Of observations with missing data on HRS covariates, 99% had missing BMI. ADRD, Alzheimer's disease and related dementias; BMI, body mass index; CCW, Chronic Conditions Data Warehouse; FFS, fee‐for‐service; HRS, Health and Retirement Study.

Data from HRS contained a maximum of 10 biennial waves over 18 years of follow‐up. A subset of the HRS nationally‐representative study of community‐dwelling older adults with available linkage to Medicare fee‐for‐service claims was utilized (*n* = 25 857). In total, our sample represents 9944 respondents with CCW linkage from 1998 to 2016, between the ages of 68 and 99, self‐reporting race and ethnicity as Hispanic, non‐Hispanic Black, or non‐Hispanic White. Participants needed to be free of ADRD based on the CCW at their first included observation; and with at least two observations (i.e., fee‐for‐service enrollment regardless of presence/absence of claims and one wave of HRS) (Figure 1).

### 2.2 ADRD

ADRD status was ascertained based on claims occurring within a 3‐year lookback period (Table S1). Based on the CCW algorithm, if at least one inpatient, skilled nursing home, home health agency, or carrier claim with an ADRD‐related ICD‐9/ICD‐10 code was recorded during the lookback period, ADRD was coded present from the date of the first calendar year being examined onward.

### 2.3 Chronic conditions

Data on eight chronic conditions, based on validated standardized claims algorithms from the CCW, were included (Table S1). For selected chronic conditions—diabetes, ischemic heart disease, STIA, AMI, atrial fibrillation, rheumatoid arthritis or osteoarthritis, heart failure, and hypertension—previous findings reported associations with dementia. The presence of chronic conditions and ADRD was coded from the first calendar year indicated by the CCW algorithm onward.^44^

Evidence for the association of diabetes and hypertension with ADRD risk has been consolidated previously, with varying magnitudes of strength and resulting PAFs.^1^, ^39^ Atrial fibrillation has been associated with an increased risk of ADRD, VaD, and AD, albeit mixed findings of AD risk in later‐life^‐^onset atrial fibrillation.^9^, ^10^ Both incident and prevalent STIAs were suggested as potentially modifiable risk factors of incident all‐cause dementia in previous studies.^12^

For arthritis, previous findings suggest an increased risk of cognitive decline and ADRD for those with rheumatoid arthritis but not for those with osteoarthritis.^13^ Despite likely differential biological pathways linking ADRD to rheumatoid arthritis or osteoarthritis (e.g., rheumatoid arthritis to coronary heart disease, or osteoarthritis to metabolic syndrome and, consequently, ADRD), we opted to include the combined category available in the CCW to capture the overall contribution of rheumatoid or osteoarthritis to ADRD incidence.^13^, ^22^, ^45^, ^46^ The role of heart failure in ADRD risk has been investigated previously but findings are less clear, likely due to the inherently complex interrelationships (heart failure and ADRD may share a common bological pathway, such as atherosclerosis; heart failure may reflect step on a cardiovascular path to ADRD, involving atrial fibrillation or hypertension, e.g.) and resulting differences in analytical strategies.^11^

Ischemic/coronary heart disease was found to be associated with cognitive impairment and VaD, but not AD, in later life.^14^ Similarly, a prior study showed that AMI was associated with cognitive decline in line with an assumed path to increased ADRD risk involving coronary heart disease.^11^, ^14^ Nonetheless, we included ischemic heart disease due to our interest in estimating the joint contribution of chronic conditions to ADRD as presented in Section 2.2.

There is a vast research landscape suggesting associations of the eight included chronic conditions and increased risk of AD, VaD, or ADRD. Further evidence suggests specific chronic condition clusters, such as cardiometabolic syndrome or cardiometabolic multimorbidity, to convey excess risk of ADRD.^16^, ^17^, ^37^ Despite a higher ADRD risk in individuals with cardiometabolic syndrome, and plausible working mechanisms linking hyperlipidemia to higher ADRD risk (e.g., by impeding blood–brain barrier integrity), we opted not to include hyperlipidemia as a risk factor due to limited evidence from randomized controlled trials (RCTs).^6^, ^47^

### 2.4 Covariates

Covariates were selected to reduce confounding based on the literature.^1^, ^3^, ^19^, ^30^, ^31^, ^32^, ^33^ Notably, older age is a well‐known predictor of ADRD; women have a higher incidence than men; minoritized adults have a higher incidence than non‐Hispanic White adults; coupled status has shown differing effects for wives and husbands; greater net worth and higher education are indicators for access to health care and health literacy and appear protective for ADRD; higher body mass index (BMI) is a risk factor for many chronic conditions, while the reported BMI paradox with dementia must be considered; and disability in activities of daily living can be the result of chronic conditions and precede dementia diagnosis.^48^

Age was calculated as the difference between the year that a participant entered the study and their birth year. Because we modeled relative risk over person‐years, with participants entering the study at different ages, models were adjusted for median age at baseline. Both age (age >76 years, yes vs no) and net worth (net worth >$211 000, yes vs no) were dichotomized at their median value. Sex (male vs female), race (Black vs White), ethnicity (Hispanic vs non‐Hispanic), and coupled status (married/partnered vs not married/partnered) were self‐reported. Additional sociodemographic factors were dichotomized including education (less than 12 vs 12 or more years of education), BMI (less than/equal 30 vs BMI greater 30), and limitations with activities of daily living (ADL; less than two ADL vs. two or more ADL). Net worth, BMI, and ADL were updated per HRS wave.

### 2.5 Statistical analysis

For analyses, observations were left‐truncated at age 68 due to eligibility to enter Medicare at age 65 and incorporating a 3‐year lookback period required to apply the ADRD CCW algorithm. Individuals were right‐censored after their incident ADRD diagnosis, or at the end of the study in 2016, or above age 99, or at their first missing data from the Centers for Medicare & Medicaid Services (i.e., if participants changed enrollment) or HRS (i.e., in case of non‐participation or item non‐response for any of the three time‐varying covariates: net worth, BMI, and ADL), whichever occurred first. Censoring individuals at their change in Medicare enrollment was conducted to avoid differential inclusion of participants based on their health outcomes at re‐entry into fee‐for‐service Medicare plans.^43^

Sociodemographic characteristics from HRS were merged with CCW data containing chronic conditions and ADRD codes. Because CCW codes are determined annually but HRS is a biennial survey, HRS data were carried forward for the subsequent year. As such, only complete observations of participants, that is, observations while enrolled in fee‐for‐service and without missing HRS covariates were included in the analytic data set.

PAFs reflect the percentage of ADRD cases that are due to the presence of the chronic condition, assuming a causal relationship. Previously employed approaches generally relied on the PAF formula based on prevalence data and a variety of relative measures of association.^2^, ^28^, ^29^, ^31^, ^39^, ^49^

LE‐AAFs reflect the average proportion of ADRD attributable to a chronic condition when considering the complete follow‐up time, coexisting conditions, their multiple combinations, and participant characteristics. Thus, this method extends on commonly employed approaches to estimate PAFs that are based on aggregated (adjusted) relative measures of risk.^28^, ^29^, ^40^, ^41^, ^49^ The procedures have estimate LE‐AAFs have been described earlier.^41^, ^42^

Briefly, a pooled logistic regression model is specified with observations reflecting individual years per participant, with yearly chronic conditions, binary covariates, and continuous time (in years) as predictors, and ADRD as outcome. Covariates were chosen to allow the estimation of a direct effect of chronic conditions with ADRD. Individual follow‐up is computed as the time in years from the time participants became eligible (e.g., age 68 or older, first observation) to the end of follow‐up (e.g., first year with ADRD code, age 99, or censoring).

To derive LE‐AAF for an individual chronic condition, say hypertension, adjusted attributable fractions (AAFs) for each timepoint (i.e., year) are computed first. To compute the AAF for hypertension, coefficients reflecting the association of hypertension with ADRD, resulting from the pooled logistic regression, are used to compute the probability of dementia (A) with and (B) without hypertension (Box S1). The difference in the probability of ADRD (A) with and (B) without hypertension is then multiplied with the prevalence of hypertension, divided by the overall probability of ADRD at the specific timepoint (i.e., year).

Given eight chronic conditions, participants fall into one of 2^8^ strata, with variation over time. For example, participants may develop hypertension first, then, two years later, stroke, but remain unaffected by further comorbid conditions. To account for multiple sequences, probabilities of ADRD with and without hypertension are computed for every stratum *k*, defined by presence or absence of comorbid conditions, at every observed time *t* (i.e., based on observations from participants at a specific year, Box S1). Critically, probabilities of having ADRD in (A) with and (B) without hypertension will differ based on the removal order of conditions, that is, the presence/absence of conditions constituting *k*.^34^, ^50^, ^51^ As the aim of LE‐AAF is to provide a population‐level estimate, sequential AAFs for every observed ordering are computed, and then averaged over all observed removal orders/sequences to retrieve an average AAF for hypertension at *t* ^34^, ^41^, ^50^, ^51^ (see Box S1 and Figure S1, e.g., computation).

The weighted average of AAF of hypertension at times *t_i_* {*i* ∈ ℝ: 0 ≤ *i* ≤ 18}, with weights reflecting person‐years, then denotes the LE‐AAF estimate for hypertension. Thus the temporal proximity to the outcome ADRD is established as the time individuals are exposed to risk factors in a given stratum, that is, in isolation or with comorbid conditions, as reflected in weighting.^41^ Moreover, deriving LE‐AAF from AAF establishes component‐additivity and, as such, enforces the total sum of individual LE‐AAFs to reflect the combined attributable risk over all LE‐AAFs.^50^, ^51^, ^52^ Component‐additivity of the LE‐AAF method constitutes an advantage over standard PAF that may sum to exceed their joint contribution due to ignoring multi‐exposed individuals.^53^, ^54^

LE‐AAFs for each chronic condition are computed with bias‐corrected and accelerated bootstrap intervals (BCas). Specifically, BCas utilize bias (i.e., the proportion of bootstrapped LE‐AAF estimates lower than the LE‐AAF estimate based on the complete sample) and acceleration (based on the difference of jackknifed LE‐AAF estimates to the mean of all jackknifed LE‐AAF estimates) to adjust percentiles denoting the lower and upper bounds of the confidence interval (CI), given the distribution of bootstrapped LE‐AAF estimates.^55^ BCas were computed over 3000 bootstrap samples to estimate 95% CIs.^55^

Sensitivity analyses comprised comparison of LE‐AAF estimates to PAFs based on Miettinen's formula using adjusted odds ratios (PAF_ORs_) and unadjusted risk ratios (PAF_RRs_). Furthermore, estimates from pooled logistic regressions were compared to estimates based on data from participants excluded from primary analyses due to missing information in HRS covariate data, or change in Medicare fee‐for‐service enrollment or to the full data with an indicator for missingness.

Example SAS code for LE‐AAF and BCa computation is available online as is code for analyses presented in this article.^56^, ^57^ All analyses were conducted with SAS v.9.4 (SAS Institute, Cary, NC).

## 3 RESULTS

After application of eligibility and censoring criteria, data from *n* = 9944 participants (mean [SD] age at baseline = 72.8 [6.5] years) were included in analyses. Most participants self‐reported being female (57.6%), White (88.3%), and of non‐Hispanic ethnicity (94.3%). In total, *n* = 2027 (20.4%) of the sample met the CCW ADRD definition over the course of the study (median interquartile range [IQR] = 6^3^, ^4^, ^5^, ^6^, ^7^, ^8^, ^9^, ^10^ years of follow‐up). Descriptive characteristics are shown in Table 1 (see Table S2 for participants excluded from primary analyses).

**TABLE 1:** Participant characteristics at baseline (total *n* = 9944).

Characteristic	
Age in years, mean (SD)	72.8 (6.5)
Female, *n* (%)	5732 (57.6%)
Race/ethnicity	
Black, *n* (%)	1163 (11.7%)
Hispanic, *n* (%)	566 (5.7%)
Twelve or more years of education, n (%)	6916 (69.5%)
Coupled, *n* (%)	6365 (64.0%)
Net worth, median (IQR)	$168 050 ($51 000, $435 000)
Body mass index, mean (SD)	27.1 (5.3)
Two or more ADL limitations, *n* (%)	889 (8.9%)

Abbreviations: ADLs, activities of daily living; IQR, interquartile range; SD, standard deviation.

**FIGURE 2:**
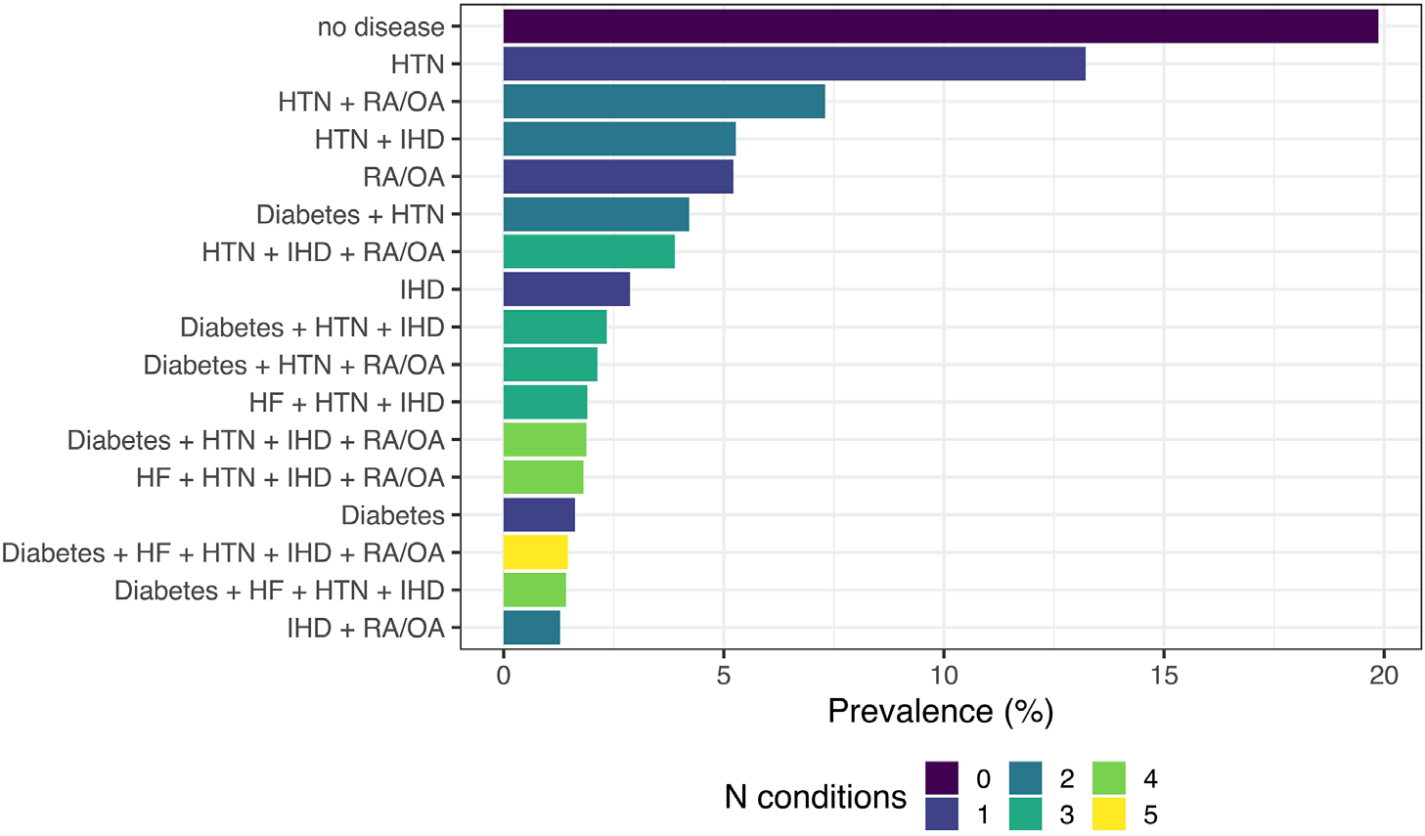
Prevalence of cardiometabolic multimorbidity combinations at baseline. Note that prevalence combinations ocurring in less than 1% of the sample are not depicted. AFIB, atrial fibrillation; AMI, acute myocardial infarction; HF, heart failure; HTN, hypertension; IHD, ischemic heart disease; RA/OA, rheumatoid arthritis or osteoarthritis; STIA, stroke or transient ischemic attack.

Chronic conditions were highly prevalent. Only 19.9% of the sample had no chronic condition at baseline (Table 2). Hypertension was the most prevalent chronic condition (63.0% of participants had hypertension at baseline). This was also true in participants excluded from the primary analyses (Table S3). Of 256 possible chronic condition combinations, 160 were observed at baseline (Table 3). Note that AMI was the only condition that did not occur in isolation at baseline. Figure 2 illustrates the prevalence of mutually exclusive disease combinations at baseline. Although the largest prevalence is denoted for “no disease” at baseline, hypertension is the most common individual chronic condition and most frequent constituent of chronic condition combinations.

**TABLE 2:** Distribution of chronic conditions at baseline.

Characteristic	*n* (%)
Acute myocardial infarction	322 (3.2%)
Atrial fibrillation	758 (7.6%)
Diabetes	2265 (22.8%)
Heart failure	1885 (19.0%)
Hypertension	6269 (63.0%)
Ischemic heart disease	3776 (38.0%)
Rheumatoid or osteoarthritis	3465 (34.8%)
Stroke or transient ischemic attack	839 (8.4%)
Number of conditions at baseline	
0	1976 (19.9%)
1	2371 (23.8%)
2	2269 (22.8%)
3	1609 (16.2%)
4	1004 (10.1%)
5	505 (5.1%)
6+	210 (2.1%)

**TABLE 3:** Unique combinations of chronic conditions at baseline.

	Number of unique combinations
Total combinations (including no conditions)	160
According to number of conditions	
0	1
1	7^a^
2	22
3	36
4	42
5	31
6+	21

^a^ At baseline, all participants with acute myocardial infarction had more than one chronic condition.

All chronic conditions were significantly associated with increased odds of ADRD in the pooled logistic regression, except AMI (OR = 1.14, 95% CI = 0.98–1.32). Odds of ADRD were further higher with increasing years since baseline (OR = 1.08, 95% CI = 1.07–1.09), above median age at baseline (OR = 2.21, 95% CI = 1.99–2.44]), two or more activities of daily living (ADL) limitations (OR = 1.76, 95% CI = 1.57–1.98), and Black race (OR = 1.22, 95% CI = 1.06–1.42). There was no association of ADRD with female sex (OR = 1.05, 95% CI = 0.95–1.17), coupled status (OR = 0.96, 95% CI = 0.86–1.06), or Hispanic ethnicity (OR = 1.19, 95% CI = 0.97–1.45). A higher BMI was associated with a lower odds of ADRD (OR = 0.55, 95% CI = 0.49–0.63) as was higher education (OR = 0.89, 95% CI = 0.80–0.99) and above median net worth (OR = 0.81, 95% CI = 0.73–0.90) (Table 4).

**TABLE 4:** Model coefficients from pooled logistic regression.

Variable	Estimate	SE	OR (95% CI)	*p* value
Time	0.0761	0.006	1.08 (1.07, 1.09)	<0.001
Acute myocardial infarction	0.127	0.077	1.14 (0.98, 1.32)	0.10
Atrial fibrillation	0.184	0.057	1.2 (1.08, 1.34)	0.001
Diabetes	0.133	0.050	1.14 (1.04, 1.26)	0.008
Heart failure	0.384	0.054	1.47 (1.32, 1.63)	<0.001
Hypertension	0.241	0.079	1.27 (1.09, 1.49)	0.002
Ischemic heart disease	0.199	0.057	1.22 (1.09, 1.37)	<0.001
Rheumatoid or osteoarthritis	0.246	0.053	1.28 (1.15, 1.42)	<0.001
Stroke, or transient ischemic attack	0.675	0.051	1.96 (1.78, 2.17)	<0.001
Female	0.050	0.053	1.05 (0.95, 1.17)	0.34
Twelve or more years of education	−0.118	0.054	0.89 (0.8, 0.99)	0.030
Net worth over $211 000	−0.209	0.054	0.81 (0.73, 0.9)	<0.001
BMI over 30	−0.590	0.067	0.55 (0.49, 0.63)	<0.001
Coupled	−0.044	0.054	0.96 (0.86, 1.06)	0.41
Baseline age above 76	0.792	0.052	2.21 (1.99, 2.44)	<0.001
Two or more ADL limitations	0.565	0.059	1.76 (1.57, 1.98)	<0.001
Black	0.203	0.075	1.22 (1.06, 1.42)	0.007
Hispanic	0.172	0.102	1.19 (0.97, 1.45)	0.09

Abbreviations: ADLs, activities of daily living; BMI, body mass index; OR, odds ratio; SE, standard error.

**FIGURE 3:**
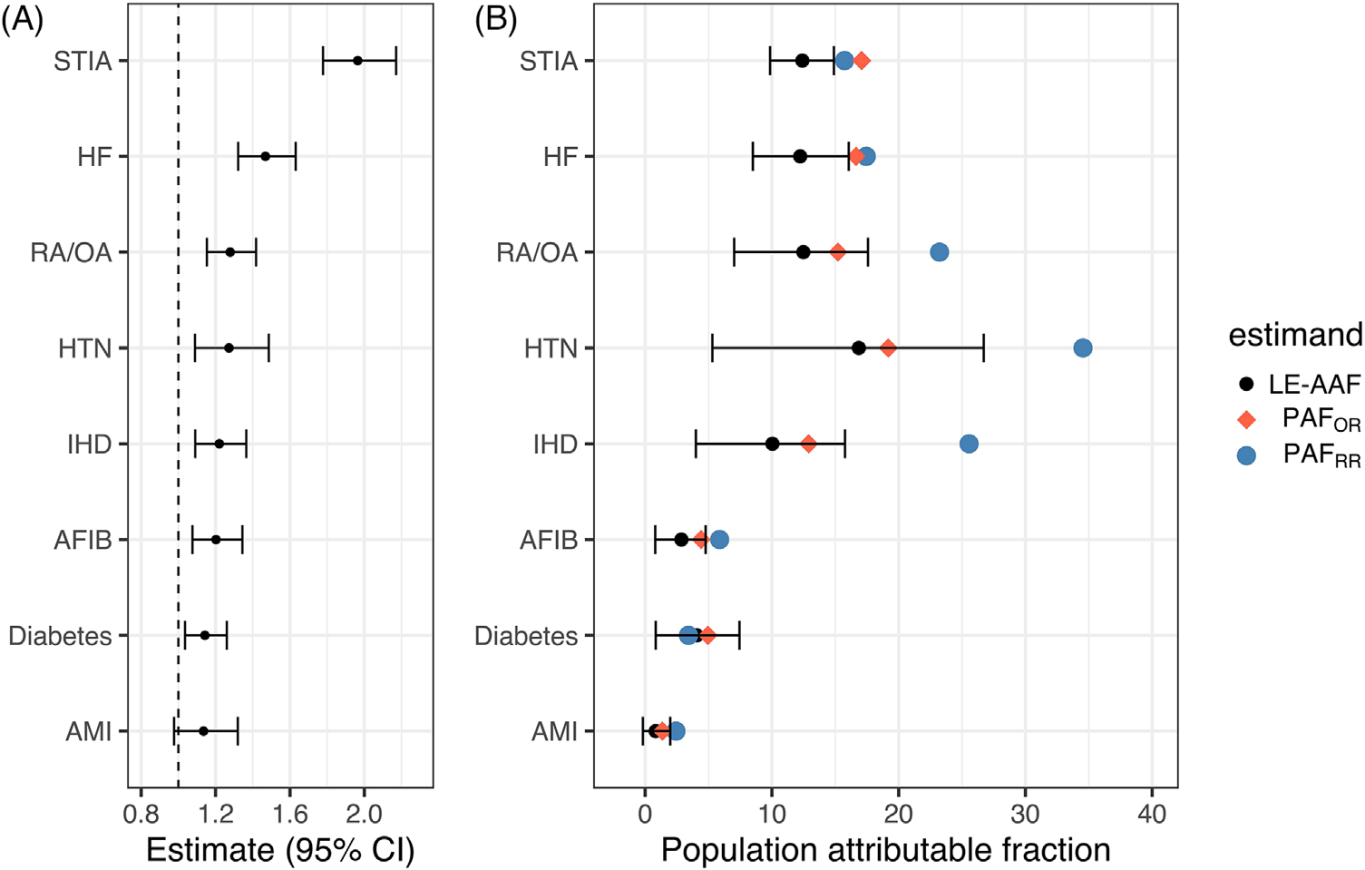
(A) Odds ratios with 95% CIs from the pooled logistic regression. (B) LE‐AAF estimates with 95% BCa CIs (black circles and error bars), PAFs based on PAF_ORs_ (red diamonds) and PAF_RRs_ (blue circles). AFIB, atrial fibrillation; AMI, acute myocardial infarction; HF, heart failure; HTN, hypertension; IHD, ischemic heart disease; PAFs, population attributable fractions; PAF_ORs_, adjusted odds ratios; PAF_RRs_, unadjusted risk ratios; RA/OA, rheumatoid arthritis or osteoarthritis; STIA, stroke or transient ischemic attack.

All LE‐AAFs for chronic conditions suggested significant contributions to ADRD incidence, except for AMI (Figure 3). For AMI, there was a positive but non‐significant contribution to ADRD incidence (LE‐AAF [95% BCa CI] = 0.8% [0.2%, 2.0%]).

LE‐AAF was highest for hypertension (LE‐AAF [95% BCa CI] = 16.9% [5.3%, 26.7%]). Related constituents of a cardiovascular path to ADRD, involving hypertension, were strongly contributing to ADRD incidence (e.g., STIA, LE‐AAF [95% BCa CI] = 12.4% [9.9%, 14.9%]).

LE‐AAF analysis suggested 71.8% (95% BCa CI [62.9%, 79.1%]) of ADRD cases in this study can be attributed to the covariate‐adjusted association of eight chronic conditions under investigation (Table 5).

**TABLE 5 alz14589-tbl-0005:** Estimates from the longitudinal extension of the average attributable fraction (LE‐AAF).

Condition	LE‐AAF (95% BCA CI)
Acute myocardial infarction	0.8 (−0.2, 2.0)
Atrial fibrillation	2.9 (0.8, 4.8)
Diabetes	4.1 (0.8, 7.4)
Heart failure	12.2 (8.5, 16.1)
Hypertension	16.9 (5.3, 26.7)
Ischemic heart disease	10.0 (4.0, 15.8)
Rheumatoid arthritis or osteoarthritis	12.5 (7.0, 17.6)
Stroke or transient ischemic attack	12.4 (9.9, 14.9)
Total	71.8 (62.9, 79.1)

Abbreviation: BCA, Bias‐corrected and accelerated bootstrap intervals over 3000 bootstrap samples; LE‐AAF, longitudinal extension of the average attributable fraction.

Sensitivity analysis of pooled logistic regression models in participants excluded from the primary analyses due to missing HRS covariate data or change in Medicare fee‐for‐service enrollment suggests similar point estimates despite wider CIs compared to the primary analysis (Figure S2). Similarly, pooled logistic regression in the full sample including participants from primary and sensitivity analyses, with an indicator of study eligibility, suggested similar point estimates but reduced odds of ADRD given eligibility (Figure S2).

Further sensitivity analyses PAF based on Miettinen's formula using adjusted PAF_ORs_ and PAF_RRs_ (Figure 3, Box S2, and Table S4) suggest internal validity of LE‐AAFs.^29^, ^49^ Except for STIA and heart failure, PAF_ORs_ were within 95% BCa CI of computed LE‐AAFs. PAFs were generally higher than LE‐AAFs, also for adjusted (PAF_OR_), but more so for unadjusted PAF_RR_ and highly prevalent chronic conditions such as hypertension, rheumatoid arthritis or osteoarthritis, and ischemic heart disease.

## 4 DISCUSSION

We estimated the absolute contribution of chronic conditions to dementia incidence, accounting for multimorbidity co‐evolution over time. Over two‐thirds (71.8%) of dementia cases among Medicare fee‐for‐service beneficiaries were attributable to hypertension, AMI, atrial fibrillation, diabetes, heart failure, ischemic heart disease, STIA, and rheumatoid arthritis or osteoarthritis. Contributions to ADRD incidence were highest for hypertension and other conditions constituting a vascular pathway to ADRD.

### 4.1 General discussion

Prior work investigating multifactorial inter‐relationships between chronic conditions and dementia incidence has fallen short of addressing multimorbidity over time. Studies examining ADRD risk of chronic conditions focused on estimating multiply‐adjusted measures of RR for individual chronic conditions or preselected chronic condition combinations at a specific time.^17^, ^36^ These report average associations across common chronic condition patterns. Conversely, LE‐AAF estimates reflect ADRD case reduction in the population, regardless of nominal comorbidity groups or sequences.^34^

Of note, the joint contribution is based on associations estimated in a covariate‐adjusted pooled logistic regression model. Hence, the unexplained contributions could vary based on the inclusion of further conditions or characteristics associated with ADRD. We extend prior findings by averaging across multimorbidity patterns over time, thereby providing estimates of the absolute contribution of chronic conditions to population‐level ADRD. The LE‐AAFs presented may better inform public health stakeholders and guide clinical practice than previous approaches due to their generalizability across multimorbidity patterns and ability to account for follow‐up time.

The greatest contribution of an individual chronic condition to ADRD was found for hypertension, approaching 20%. Although this accords with previous studies that adjusted PAFs of hypertension for comorbid conditions, our findings reinforce the importance of managing hypertension. LE‐AAFs suggest that the management of hypertension may decrease ADRD incidence, irrespective of and in addition to potentially beneficial effects of managing potentially co‐occurring chronic conditions.^32^, ^39^ This generalizability is a marked difference to previously reported PAFs and of importance given the accelerated accumulation of multimorbidity as early as 20 years prior to impaired cognition in dementia and related spillover effects.^8^ Our findings point to the additive contributions of the examined conditions for ADRD risk in the general U.S. population and add to the extant literature suggesting modifiability of ADRD risk.^58^ As the LE‐AAF combines the prevalence, incidence, and associations of conditions, this suggests that the impact of reducing the incidence and prevalence of individual long‐term chronic conditions may, in turn, reduce the level of ADRD in the population. Although this approach incorporates temporality, we cannot rule out that in some cases ADRD may have preceded the included long‐term chronic conditions, given the interval spacing of the HRS data collection waves.[Table alz14589-tbl-0005]

The reported LE‐AAF for STIA aligns with previous evidence on detriments to cognitive function and increased dementia risk following STIA, particularly when of hemorrhagic nature.^12^, ^15^, ^19^, ^59^ Contributions of heart failure and ischemic heart disease to ADRD suggest relative importance of pathways involving hypoperfusion and hallmark indicators of AD.^11^ Accounting for concomitant heart failure may further explain the modest significant contribution of atrial fibrillation and related neuronal injury to ADRD incidence.^9^, ^10^, ^60^ Pathways potentially underlying the contribution of osteoarthritis with AD have been suggested by previous reports of accelerated amyloid accumulation, consequent tau deposition, and altered hippocampal functional connectivity in osteoarthritis.^61^, ^62^, ^63^ Working mechanisms for rheumatoid arthritis are less clear; however, potential drivers could be reflected in systemic inflammation and increased levels of C‐reactive protein leading to neurodegenerative progression.^22^

Although we could not separately analyze dementia subtypes, we found evidence of previously established disease trajectories contributing to an overwhelming fraction of dementia cases in the United States. Of note, additive contributions of hypertension, STIA, heart failure, and ischemic heart disease make up for two‐thirds of the joint LE‐AAF. Given VaD as the second most common form of dementia and, for example, small vessel injury due to hypertension as a strong risk factor of AD, our results reinforce the population‐level contribution of longstanding hypertension to all‐cause dementia and suggest accumulation of downstream vascular damage as a primary target for population‐level dementia prevention.^19^

LE‐AAFs, like PAFs, are larger for more prevalent, stronger associated and timely‐related risk factors. In our sensitivity analysis, conventionally estimated PAFs were higher than previously reported and generally exceeded LE‐AAFs. Given the non‐additivity of conventionally estimated PAFs, joint contributions appear overestimated. Because LE‐AAFs were based on the same underlying log odds, comparison with PAF_OR_ suggests that LE‐AAFs are less prone to overestimating the contribution of individual chronic conditions to ADRD risk, and that emerging differences are due to averaging across multimorbidity sequences and weighting for time at‐risk. Of note, except for STIA and heart failure, PAF_ORs_ fall within the 95% BCa CI; hence, they do not differ statistically from LE‐AAFs. As such, LE‐AAFs reported in this study provide a more complete picture of the absolute, additive contribution of chronic conditions to dementia considering synergies in present multimorbidity.

The prevalence of chronic conditions were similar to previously reported population‐level estimates while differing compared with previous studies estimating PAFs.^64^ ORs were similar to previously reported estimates and robust to sensitivity analyses in participants excluded from primary analyses.^39^, ^64^ Thus, the updated relevance of individual chronic conditions reported in this study may not reflect differences in prevalence or relative risk but rather stem from adequate modeling of multimorbidity patterns over time.

At the core of this study, we find a larger joint contribution of chronic conditions to ADRD than reported previously.^1^, ^30^, ^33^, ^65^, ^66^ This accords with a study that indicated underestimation of joint contributions to ADRD risk in previous reports due to the strategies to account for communality.^35^ However, differences may result from the set of chronic conditions. Although we did not exhaustively include lifestyle‐related risk factors, we adjusted for race, ethnicity, education, net worth, ADLs, and BMI. Hence, reported LE‐AAFs are covariate‐adjusted marginal contributions to ADRD. It is important to note that later‐life physical inactivity and exposure to air pollution have reported PAFs of 1.6% and 2.3%, respectively, for ADRD.^1^, ^67^ Although we could not adjust for these, we included the presence of >2 ADLs, net worth, education, race, and ethnicity, which may be indicators for access to residential communities with better or worse air quality. Our findings may capture, in part, contributions of well‐established modifiable risk factors of dementia.

Contrary to previous reports, we found no significant contribution of AMI to ADRD incidence. Given that AMI was the only condition that did not occur in isolation at baseline, and participants needed to have survived for at least 2 years, strong associations of comorbid conditions over longer periods of follow‐up may have led to decreased contributions of AMI to ADRD incidence considering multimorbidity patterns observed over time.

We observed varying degrees of precision regarding 95% BCa CIs. The widest interval was found for hypertension. The severity of chronic conditions was not measured. Although a previous study found similar PAFs of hypertension when including potential intermediate constituents of a cardiovascular path to ADRD, the presence/absence of STIA alters computation of probabilities used to derive LE‐AAFs for, example, hypertension.^39^ Hence, the average sequential attributable fractions vary depending on the prevalence of comorbid STIA in bootstrap samples. A previous meta‐analysis suggested a more marked association of stroke than transient ischemic attacks with cognitive aging (n.s. for myocardial infarction), and further variation by type of stroke (i.e., hemorrhagic vs ischemic), which may explain the limited precision in chronic conditions such as STIA.^15^ The strength of association of chronic conditions, such as hypertension, with ADRD risk may vary according to other person characteristics (e.g., genetic predisposition, onset in mid‐ vs later life, or sex) or subtype, and hence alter LE‐AAFs.^16^, ^23^, ^68^, ^69^, ^70^

### 4.2 Strengths and limitations

In this study we employed a novel method to estimate the combined longitudinal, absolute contribution of eight chronic conditions to ADRD incidence, in a large cohort with survey data and linked health records.

Some limitations exist. The reported estimates do not provide causal evidence. Thus future research needs to examine the implied preventive potential. Moreover, with age and time restrictions, LE‐AAFs may not be interpreted as an individual‐level estimate of risk. LE‐AAFs may differ according to health care practices internationally. Participants’ Medicare enrollment may differ systematically by unobserved variables, thereby potentially biasing LE‐AAF estimates. Although no race or ethnic subgroup analysis was possible due to the limited number of participants with ADRD over time across strata, using nationally‐representative data adjusting for race and ethnicity resulted in LE‐AAF estimates generalizable to Medicare enrollees. Previous studies have suggested larger individual and sum total PAFs for Black participants.^31^, ^38^ Furthermore, left truncation before 1998 or age 68 may bias relative risk upward due to decreased time to ADRD. As LE‐AAF is restricted to binary covariates, we adjusted for median age at baseline, potentially biasing LE‐AAF upward due to underestimating the association of age with ADRD. Furthermore, chronic conditions may contribute to the semi‐competing risk of death, thus biasing LE‐AAFs downward.^40^ Finally, sensitivity and consistency of case ascertainment may vary across chronic conditions, although validation for ADRD ascertainment with a claims‐based algorithm and a 3‐year lookback period suggest maximized sensitivity with a reasonable positive predictive value.^71^, ^72^, ^73^ Use of a validated claims‐based algorithm may have misclassification or underdiagnosis for these Medicare fee‐for‐service beneficiaries.

## 5 CONCLUSION

We extend findings reporting considerable PAFs of individual and co‐occurring chronic conditions, as well as increased dementia risk in multimorbidity by using updated methodology accounting for comorbidity patterns over time. Our findings suggest even greater potential to reduce dementia prevalence than reported previously, with a combined contribution of eight chronic conditions accounting for up to 71.8% of dementia cases. The importance of our findings is reinforced given secular trends that may increase the number of people living with ADRD by altering the prevalence of chronic conditions.^54^, ^55^, ^56^ Targeting chronic conditions on a cardiovascular path to dementia may contribute to lowering the number of individuals living with dementia, which is to be validated in future studies.

## CONFLICT OF INTEREST STATEMENT

The authors declare no conflicts of interest. Author disclosures are available in the Supporting Information.

## CONSENT STATEMENT

The Oregon Health and Science University Research Integrity Office Institutional Review Board has exempted the need to obtain informed consent and has provided a waiver to use the information in secondary analysis (STUDY00019414). Methods and analyses comply with the relevant guidelines and regulations.

## DIVERSITY, EQUITY, AND INCLUSION STATEMENT

Although the Health and Retirement Study (HRS) provides population‐based data, limited diversity in the subset with available Medicare linkage did not allow for stratified analyses. We discuss consequences in detail in the limitations. Diversity was instead addressed by adjusting models for proxies of race and ethnicity.

## ETHIC STATEMENT

This study received approval from the institutional review board of the Oregon Health & Science University (IRB ID# STUDY00019414).

## Supporting information



Supporting information

Supporting information
