# Supplementary material for: Examining multimorbidity contributors to dementia over time
Source: Alzheimers Dement. 2025 Feb 23;21(2):e14589. doi: 10.1002/alz.14589 (PMC11847647; doi:10.1002/alz.14589)
Supplement: Supplementary file 1 — Supporting information [file ALZ-21-e14589-s002.docx]

**Examining dementia fraction attributable to multimorbidity over time – Supplementary Material**

**References**

1. Allore HG, Zhan Y, Cohen AB, Tinetti ME, Trentalange M, McAvay G. Methodology to Estimate the Longitudinal Average Attributable Fraction of Guideline-recommended Medications for Death in Older Adults With Multiple Chronic Conditions. *J Gerontol A Biol Sci Med Sci*. 2016;71(8):1113-1116. doi:10.1093/gerona/glv223

2. Lin H, Allore HG, McAvay G, et al. A Method for Partitioning the Attributable Fraction of Multiple Time-Dependent Coexisting Risk Factors for an Adverse Health Outcome. *Am J Public Health*. 2013;103(1):177-182. doi:10.2105/AJPH.2011.300458

3. Murphy TE, McAvay G, Carriero NJ, et al. Deaths observed in Medicare beneficiaries: average attributable fraction and its longitudinal extension for many diseases. *Stat Med*. 2012;31(27):3313-3319. doi:10.1002/sim.5337

4. Eide GE, Gefeller O. Sequential and average attributable fractions as aids in the selection of preventive strategies. *J Clin Epidemiol*. 1995;48(5):645-655. doi:10.1016/0895-4356(94)00161-I

5. Gefeller O, Land M, Eide GE. Averaging Attributable Fractions in the Multifactorial Situation: Assumptions and Interpretation. *J Clin Epidemiol*. 1998;51(5):437-441. doi:10.1016/S0895-4356(98)00002-X

6. Zhan Y, McAvay G. Longitudinal Extension of the Average Attributable Fraction (LE-AAF). Geriatric Research Algorithms & Statistical Programs (GRASP). Accessed November 30, 2024. https://www.peppercenter.org/public/grasp.cfm

7. Lin CK, Chen ST. Estimation and application of population attributable fraction in ecological studies. *Environ Health*. 2019;18(1):52. doi:10.1186/s12940-019-0492-4

8. Miettinen OS. Proportion of disease caused or prevented by a given exposure, trait or intervention. *Am J Epidemiol*. 1974;99(5):325-332. doi:10.1093/oxfordjournals.aje.a121617

9. Di Maso M, Bravi F, Polesel J, et al. Attributable fraction for multiple risk factors: Methods, interpretations, and examples. *Stat Methods Med Res*. 2020;29(3):854-865. doi:10.1177/0962280219848471

10. MacMahon B, Pugh TF. Epidemiology: principles and methods. Published online 1970.

**Box S1.** Example computation of the adjusted attributable fraction for hypertension in stratum k at timepoint t.

1. Run pooled logistic regression with ADRD as outcome and chronic conditions as predictors (adjusted for covariates and continuous time), over the complete set of observations (i.e., person-years)

For every person-year:

For every stratum *k:*

1. Compute adjusted attributable fraction (AF) for hypertension in stratum *k_H/D/S_* with hypertension, with diabetes and stroke, at time *t*
   1. Compute probability of ADRD (A) with/(B) without hypertension based on coefficients from the pooled logistic regression

*A = 1 / [1 + exp { - ( β_0_ + 1 * β_1 HTN_ + 1 * β_2 Diabetes_ + 1 * β_3 Stroke_) } ]* (1)

*B = 1 / [1 + exp { - ( β_0_  + 0 * β_1 HTN_ + 1 * β_2 Diabetes_ + 1 * β_3 Stroke_ ) } ]* (2)

- 1. Compute AF for hypertension

*AF_H/D/S_ = [A – B] x (p_H_ / P_ADRD_)* (3)

*With p_H_ = Prevalence of hypertension in k_H/D/S_*

*P_ADRD_ = Probability of ADRD at t*

- 1. Repeat for every stratum *k*, removing one condition at a time, in all possible orders

1. Compute sequential adjusted attributable fraction (SAF) for hypertension at time *t*
   1. compute SAF for hypertension as proportional reduction in incident ADRD, comparing AF for different *k*
      e.g., if hypertension would have been removed first, the difference of AFs for stratum *k_H/D/S_* to *k_D/S_* with diabetes and stroke but without hypertension

*SAF_(H)/D/S_ = AF_H/D/S_ - AF_D/S_* (4)

- 1. Repeat to compute SAF for all removal orders

1. Compute the average attributable fraction (AAF) by averaging across all possible SAF at time *t*
2. Compute weighted average of AAF for all *t* with weights according to person-years

Greater details are provided in previous publications ^1–5^. SAS code, instructions and BCa bootstrap code available online ^6^.

**Box S2.** Alternative formulations of population attributable fractions.

Miettinen's formula:

PAF = p * ((RR - 1) / RR) (5)
where p is the prevalence of hypertension (exposed) in participants with dementia

Probability based formulation:

PAF = P(HTN|ADRD) * (1-RR^-1^) (6)
where P(HTN|ADRD) is the prevalence of hypertension (exposed) in participants with dementia

Miettinen’s formula (5) may be expressed in terms of probabilities (6) using Bayes theorem and MacMahon and Pugh's formula of the population attributable fraction (PAF) ^7–10^. Note that (6) compares to (3) in that the PAF is computed as the product of the probability of dementia given presence/absence of a risk factor (described as RR in (6) and as probability in (3)) divided by the risk of dementia (described as RR in (6) and as probability in (3)).

Note that Miettinen’s formula based on adjusted odds ratios instead of risk ratios, was utilized to test sensitivity of LE-AAF in the present study. As such, we computed PAFs (based on unadjusted risk ratios or adjusted odds ratios) to compare LE-AAF, which are based on identical coefficients from the pooled logistic regression to PAFs (5) that are agnostic to time to ADRD and sequences of condition onset. Of note, adjusted odds ratios may overestimate relative risk as ADRD is not rare in this sample with ~ 20.4%. This is a limitation of our sensitivity analyses.

**Table S1.** Algorithmic classification of chronic cardiometabolic conditions.

| **Condition** | **Ref. Period (years)** | **Valid ICD-9 codes** | **Valid ICD-10 codes** | **Required Claims** |
| --- | --- | --- | --- | --- |
| **AMI** | 1 | 410.01, 410.11, 410.21, 410.31, 410.41, 410.51, 410.61, 410.71, 410.81, 410.91 as first or second DX on claim) | I21.01, I21.02, I21.09, I21.11, I21.19, I21.21, I21.29, I21.3, I21.4, I21.9, I21.A1, I21.A9, I22.0, I22.1, I22.2, I22.8, I22.9 as first or second DX on claim | >= 1 inpatient claim with DX code |
| **AFIB** | 1 | 427.31 as first or second DX on claim | I48.0, I48.1, I48.11, I48.19, I48.2, I48.20, I48.21, I48.91 as first or second DX on claim | >= 1 inpatient claim **OR** 2 HOP / Carrier claims with DX codes |
| **Diabetes** | 2 | 249.00, 249.01, 249.10, 249.11, 249.20, 249.21, 249.30, 249.31, 249.40, 249.41, 249.50, 249.51, 249.60, 249.61, 249.70, 249.71, 249.80, 249.81, 249.90, 249.91, 250.00, 250.01, 250.02, 250.03, 250.10, 250.11, 250.12, 250.13, 250.20, 250.21, 250.22, 250.23, 250.30, 250.31, 250.32, 250.33, 250.40, 250.41, 250.42, 250.43, 250.50, 250.51, 250.52, 250.53, 250.60, 250.61, 250.62, 250.63, 250.70, 250.71, 250.72, 250.73, 250.80, 250.81, 250.82, 250.83, 250.90, 250.91, 250.92, 250.93, 357.2, 362.01, 362.02, 362.03, 362.04, 362.05, 362.06, 366.41 | E08.00, E08.01, E08.10, E08.11, E08.21, E08.22, E08.29, E08.311, E08.319, E08.321, E08.3211, E08.3212, E08.3213, E08.3219, E08.329, E08.3291, E08.3292, E08.3293, E08.3299, E08.331, E08.3311, E08.3312, E08.3313, E08.3319, E08.339, E08.3391, E08.3392, E08.3393, E08.3399, E08.341, E08.3411, E08.3412, E08.3413, E08.3419, E08.349, E08.3491, E08.3492, E08.3493, E08.3499, E08.351, E08.3511, E08.3512, E08.3513, E08.3519, E08.3521, E08.3522, E08.3523, E08.3529, E08.3531, E08.3532, E08.3533, E08.3539, E08.3541, E08.3542, E08.3543, E08.3549, E08.3551, E08.3552, E08.3553, E08.3559, E08.359, E08.3591, E08.3592, E08.3593, E08.3599, E08.36, E08.37X1, E08.37X2, E08.37X3, E08.37X9, E08.39, E08.40, E08.41, E08.42, E08.43, E08.44, E08.49, E08.51, E08.52, E08.59, E08.610, E08.618, E08.620, E08.621, E08.622, E08.628, E08.630, E08.638, E08.641, E08.649, E08.65, E08.69, E08.8, E08.9, E09.00, E09.01, E09.10, E09.11, E09.21, E09.22, E09.29, E09.311, E09.319, E09.321, E09.3211, E09.3212, E09.3213, E09.3219, E09.329, E09.3291, E09.3292, E09.3293, E09.3299, E09.331, E09.3311, E09.3312, E09.3313, E09.3319, E09.339, E09.3391, E09.3392, E09.3393, E09.3399, E09.341, E09.3411, E09.3412, E09.3413, E09.3419, E09.349, E09.3491, E09.3492, E09.3493, E09.3499, E09.351, E09.3511, E09.3512, E09.3513, E09.3519, E09.3521, E09.3522, E09.3523, E09.3529, E09.3531, E09.3532, E09.3533, E09.3539, E09.3541, E09.3542, E09.3543, E09.3549, E09.3551, E09.3552, E09.3553, E09.3559, E09.359, E09.3591, E09.3592, E09.3593, E09.3599, E09.36, E09.37X1, E09.37X2, E09.37X3, E09.37X9, E09.39, E09.40, E09.41, E09.42, E09.43, E09.44, E09.49, E09.51, E09.52, E09.59, E09.610, E09.618, E09.620, E09.621, E09.622, E09.628, E09.630, E09.638, E09.641, E09.649, E09.65, E09.69, E09.8, E09.9, E10.10, E10.11, E10.21, E10.22, E10.29, E10.311, E10.319, E10.321, E10.3211, E10.3212, E10.3213, E10.3219, E10.329, E10.3291, E10.3292, E10.3293, E10.3299, E10.331, E10.3311, E10.3312, E10.3313, E10.3319, E10.339, E10.3391, E10.3392, E10.3393, E10.3399, E10.341, E10.3411, E10.3412, E10.3413, E10.3419, E10.349, E10.3491, E10.3492, E10.3493, E10.3499, E10.351, E10.3511, E10.3512, E10.3513, E10.3519, E10.3521, E10.3522, E10.3523, E10.3529, E10.3531, E10.3532, E10.3533, E10.3539, E10.3541, E10.3542, E10.3543, E10.3549, E10.3551, E10.3552, E10.3553, E10.3559, E10.359, E10.3591, E10.3592, E10.3593, E10.3599, E10.36, E10.37X1, E10.37X2, E10.37X3, E10.37X9, E10.39, E10.40, E10.41, E10.42, E10.43, E10.44, E10.49, E10.51, E10.52, E10.59, E10.610, E10.618, E10.620, E10.621, E10.622, E10.628, E10.630, E10.638, E10.641, E10.649, E10.65, E10.69, E10.8, E10.9, E11.00, E11.01, E11.10, E11.11, E11.21, E11.22, E11.29, E11.311, E11.319, E11.321, E11.3211, E11.3212, E11.3213, E11.3219, E11.329, E11.3291, E11.3292, E11.3293, E11.3299, E11.331, E11.3311, E11.3312, E11.3313, E11.3319, E11.339, E11.3391, E11.3392, E11.3393, E11.3399, E11.341, E11.3411, E11.3412, E11.3413, E11.3419, E11.349, E11.3491, E11.3492, E11.3493, E11.3499, E11.351, E11.3511, E11.3512, E11.3513, E11.3519, E11.3521, E11.3522, E11.3523, E11.3529, E11.3531, E11.3532, E11.3533, E11.3539, E11.3541, E11.3542, E11.3543, E11.3549, E11.3551, E11.3552, E11.3553, E11.3559, E11.359, E11.3591, E11.3592, E11.3593, E11.3599, E11.36, E11.37X1, E11.37X2, E11.37X3, E11.37X9, E11.39, E11.40, E11.41, E11.42, E11.43, E11.44, E11.49, E11.51, E11.52, E11.59, E11.610, E11.618, E11.620, E11.621, E11.622, E11.628, E11.630, E11.638, E11.641, E11.649, E11.65, E11.69, E11.8, E11.9, E13.00, E13.01, E13.10, E13.11, E13.21, E13.22, E13.29, E13.311, E13.319, E13.321, E13.3211, E13.3212, E13.3213, E13.3219, E13.329, E13.3291, E13.3292, E13.3293, E13.3299, E13.331, E13.3311, E13.3312, E13.3313, E13.3319, E13.339, E13.3391, E13.3392, E13.3393, E13.3399, E13.341, E13.3411, E13.3412, E13.3413, E13.3419, E13.349, E13.3491, E13.3492, E13.3493, E13.3499, E13.351, E13.3511, E13.3512, E13.3513, E13.3519, E13.3521, E13.3522, E13.3523, E13.3529, E13.3531, E13.3532, E13.3533, E13.3539, E13.3541, E13.3542, E13.3543, E13.3549, E13.3551, E13.3552, E13.3553, E13.3559, E13.359, E13.3591, E13.3592, E13.3593, E13.3599, E13.36, E13.39, E13.40, E13.41, E13.42, E13.43, E13.44, E13.49, E13.51, E13.52, E13.59, E13.610, E13.618, E13.620, E13.621, E13.622, E13.628, E13.630, E13.638, E13.641, E13.649, E13.65, E13.69, E13.8, E13.9 | >= 1 inpatient / SNF/HHA claim **OR** 2 HOP / Carrier claims with DX codes |
| **HF** | 2 | 398.91, 402.01, 402.11, 402.91, 404.01, 404.03, 404.11, 404.13, 404.91, 404.93, 428.0, 428.1, 428.20, 428.21, 428.22, 428.23, 428.30, 428.31, 428.32, 428.33, 428.40, 428.41, 428.42, 428.43, 428.9 | I09.81, I11.0, I13.0, I13.2, I50.1, I50.20, I50.21, I50.22, I50.23, I50.30, I50.31, I50.32, I50.33, I50.40, I50.41, I50.42, I50.43, I50.810, I50.811, I50.812, I50.813, I50.814, I50.82, I50.83, I50.84, I50.89, I50.9 | >= 1 inpatient, HOP, or Carrier claim with DX code |
| **HTN** | 1 | 362.11, 401.0, 401.1, 401.9, 402.00, 402.01, 402.10, 402.11, 402.90, 402.91, 403.00, 403.01, 403.10, 403.11, 403.90, 403.91, 404.00, 404.01, 404.02, 404.03, 404.10, 404.11, 404.12, 404.13, 404.90, 404.91, 404.92, 404.93, 405.01, 405.09, 405.11, 405.19, 405.91, 405.99, 437.2 | H35.031, H35.032, H35.033, H35.039, I10, I11.0, I11.9, I12.0, I12.9, I13.0, I13.10, I13.11, I13.2, I15.0, I15.1, I15.2, I15.8, I15.9, I67.4, N26.2 | >= 1 inpatient / SNF / HHA claim **OR** 2 HOP / Carrier claims with DX codes |
| **IHD** | 2 | 410.00, 410.01, 410.02, 410.10, 410.11, 410.12, 410.20, 410.21, 410.22, 410.30, 410.31, 410.32, 410.40, 410.41, 410.42, 410.50, 410.51, 410.52, 410.60, 410.61, 410.62, 410.70, 410.71, 410.72, 410.80, 410.81, 410.82, 410.90, 410.91, 410.92, 411.0, 411.1, 411.81, 411.89, 412, 413.0, 413.1, 413.9, 414.00, 414.01, 414.02, 414.03, 414.04, 414.05, 414.06, 414.07, 414.12, 414.2, 414.3, 414.4, 414.8, 414.9 | I20.0, I20.1, I20.8, I20.9, I21.01, I21.02, I21.09, I21.11, I21.19, I21.21, I21.29, I21.3, I21.4, I21.A1, I21.A9, I22.0, I22.1, I22.2, I22.8, I22.9, I23.0, I23.1, I23.2, I23.3, I23.4, I23.5, I23.6, I23.7, I23.8, I24.0, I24.1, I24.8, I24.9, I25.10, I25.110, I25.111, I25.118, I25.119, I25.2, I25.3, I25.41, I25.42, I25.5, I25.6, I25.700, I25.701, I25.708, I25.709, I25.710, I25.711, I25.718, I25.719, I25.720, I25.721, I25.728, I25.729, I25.730, I25.731, I25.738, I25.739, I25.750, I25.751, I25.758, I25.759, I25.760, I25.761, I25.768, I25.769, I25.790, I25.791, I25.798, I25.799, I25.810, I25.811, I25.812, I25.82, I25.83, I25.84, I25.89, I25.9 | >= 1 inpatient, SNF, HHA, HOP, or Carrier claim with DX code |
| **RA/ OA** | 2 | 714.0, 714.1, 714.2, 714.30, 714.31, 714.32, 714.33, 715.00, 715.04, 715.09, 715.10, 715.11, 715.12, 715.13, 715.14, 715.15, 715.16, 715.17, 715.18, 715.20, 715.21, 715.22, 715.23, 715.24, 715.25, 715.26, 715.27, 715.28, 715.30, 715.31, 715.32, 715.33, 715.34, 715.35, 715.36, 715.37, 715.38, 715.80, 715.89, 715.90, 715.91, 715.92, 715.93, 715.94, 715.95, 715.96, 715.97, 715.98, 720.0, 721.0, 721.1, 721.2, 721.3, 721.90, 721.91 | M05.00, M05.011, M05.012, M05.019, M05.021, M05.022, M05.029, M05.031, M05.032, M05.039, M05.041, M05.042, M05.049, M05.051, M05.052, M05.059, M05.061, M05.062, M05.069, M05.071, M05.072, M05.079, M05.09, M05.20, M05.211, M05.212, M05.219, M05.221, M05.222, M05.229, M05.231, M05.232, M05.239, M05.241, M05.242, M05.249, M05.251, M05.252, M05.259, M05.261, M05.262, M05.269, M05.271, M05.272, M05.279, M05.29, M05.30, M05.311, M05.312, M05.319, M05.321, M05.322, M05.329, M05.331, M05.332, M05.339, M05.341, M05.342, M05.349, M05.351, M05.352, M05.359, M05.361, M05.362, M05.369, M05.371, M05.372, M05.379, M05.39, M05.40, M05.411, M05.412, M05.419, M05.421, M05.422, M05.429, M05.431, M05.432, M05.439, M05.441, M05.442, M05.449, M05.451, M05.452, M05.459, M05.461, M05.462, M05.469, M05.471, M05.472, M05.479, M05.49, M05.50, M05.511, M05.512, M05.519, M05.521, M05.522, M05.529, M05.531, M05.532, M05.539, M05.541, M05.542, M05.549, M05.551, M05.552, M05.559, M05.561, M05.562, M05.569, M05.571, M05.572, M05.579, M05.59, M05.60, M05.611, M05.612, M05.619, M05.621, M05.622, M05.629, M05.631, M05.632, M05.639, M05.641, M05.642, M05.649, M05.651, M05.652, M05.659, M05.661, M05.662, M05.669, M05.671, M05.672, M05.679, M05.69, M05.70, M05.711, M05.712, M05.719, M05.721, M05.722, M05.729, M05.731, M05.732, M05.739, M05.741, M05.742, M05.749, M05.751, M05.752, M05.759, M05.761, M05.762, M05.769, M05.771, M05.772, M05.779, M05.79, M05.7A, M05.80, M05.811, M05.812, M05.819, M05.821, M05.822, M05.829, M05.831, M05.832, M05.839, M05.841, M05.842, M05.849, M05.851, M05.852, M05.859, M05.861, M05.862, M05.869, M05.871, M05.872, M05.879, M05.89, M05.8A, M05.9, M06.00, M06.011, M06.012, M06.019, M06.021, M06.022, M06.029, M06.031, M06.032, M06.039, M06.041, M06.042, M06.049, M06.051, M06.052, M06.059, M06.061, M06.062, M06.069, M06.071, M06.072, M06.079, M06.08, M06.09, M06.0A, M06.1, M06.20, M06.211, M06.212, M06.219, M06.221, M06.222, M06.229, M06.231, M06.232, M06.239, M06.241, M06.242, M06.249, M06.251, M06.252, M06.259, M06.261, M06.262, M06.269, M06.271, M06.272, M06.279, M06.28, M06.29, M06.30, M06.311, M06.312, M06.319, M06.321, M06.322, M06.329, M06.331, M06.332, M06.339, M06.341, M06.342, M06.349, M06.351, M06.352, M06.359, M06.361, M06.362, M06.369, M06.371, M06.372, M06.379, M06.38, M06.39, M06.80, M06.811, M06.812, M06.819, M06.821, M06.822, M06.829, M06.831, M06.832, M06.839, M06.841, M06.842, M06.849, M06.851, M06.852, M06.859, M06.861, M06.862, M06.869, M06.871, M06.872, M06.879, M06.88, M06.89, M06.8A, M06.9, M08.00, M08.011, M08.012, M08.019, M08.021, M08.022, M08.029, M08.031, M08.032, M08.039, M08.041, M08.042, M08.049, M08.051, M08.052, M08.059, M08.061, M08.062, M08.069, M08.071, M08.072, M08.079, M08.08, M08.09, M08.0A, M08.1, M08.20, M08.211, M08.212, M08.219, M08.221, M08.222, M08.229, M08.231, M08.232, M08.239, M08.241, M08.242, M08.249, M08.251, M08.252, M08.259, M08.261, M08.262, M08.269, M08.271, M08.272, M08.279, M08.28, M08.29, M08.2A, M08.3, M08.40, M08.411, M08.412, M08.419, M08.421, M08.422, M08.429, M08.431, M08.432, M08.439, M08.441, M08.442, M08.449, M08.451, M08.452, M08.459, M08.461, M08.462, M08.469, M08.471, M08.472, M08.479, M08.48, M08.4A, M08.80, M08.811, M08.812, M08.819, M08.821, M08.822, M08.829, M08.831, M08.832, M08.839, M08.841, M08.842, M08.849, M08.851, M08.852, M08.859, M08.861, M08.862, M08.869, M08.871, M08.872, M08.879, M08.88, M08.89, M08.90, M08.911, M08.912, M08.919, M08.921, M08.922, M08.929, M08.931, M08.932, M08.939, M08.941, M08.942, M08.949, M08.951, M08.952, M08.959, M08.961, M08.962, M08.969, M08.971, M08.972, M08.979, M08.98, M08.99, M08.9A, M15.0, M15.1, M15.2, M15.3, M15.4, M15.8, M15.9, M16.0, M16.10, M16.11, M16.12, M16.2, M16.30, M16.31, M16.32, M16.4, M16.50, M16.51, M16.52, M16.6, M16.7, M16.9, M17.0, M17.10, M17.11, M17.12, M17.2, M17.30, M17.31, M17.32, M17.4, M17.5, M17.9, M18.0, M18.10, M18.11, M18.12, M18.2, M18.30, M18.31, M18.32, M18.4, M18.50, M18.51, M18.52, M18.9, M19.011, M19.012, M19.019, M19.021, M19.022, M19.029, M19.031, M19.032, M19.039, M19.041, M19.042, M19.049, M19.071, M19.072, M19.079, M19.09, M19.111, M19.112, M19.119, M19.121, M19.122, M19.129, M19.131, M19.132, M19.139, M19.141, M19.142, M19.149, M19.171, M19.172, M19.179, M19.19, M19.211, M19.212, M19.219, M19.221, M19.222, M19.229, M19.231, M19.232, M19.239, M19.241, M19.242, M19.249, M19.271, M19.272, M19.279, M19.29, M19.90, M19.91, M19.92, M19.93, M45.0, M45.1, M45.2, M45.3, M45.4, M45.5, M45.6, M45.7, M45.8, M45.9, M47.011, M47.012, M47.013, M47.014, M47.015, M47.016, M47.019, M47.021, M47.022, M47.029, M47.10, M47.11, M47.12, M47.13, M47.20, M47.21, M47.22, M47.23, M47.24, M47.25, M47.26, M47.27, M47.28, M47.811, M47.812, M47.813, M47.814, M47.815, M47.816, M47.817, M47.818, M47.819, M47.891, M47.892, M47.893, M47.894, M47.895, M47.896, M47.897, M47.898, M47.899, M47.9, M48.8X1, M48.8X2, M48.8X3, M48.8X4, M48.8X5, M48.8X6, M48.8X7, M48.8X8, M48.8X9 | >= 2 inpatient, SNF, HHA, HOP, or Carrier claims with DX codes |
| **STIA** | 1 | 430, 431, 433.01, 433.11, 433.21, 433.31, 433.81, 433.91, 434.00, 434.01, 434.10, 434.11, 434.90, 434.91, 435.0, 435.1, 435.3, 435.8, 435.9, 436, 997.02  Exclusion criteria: any 800 - 804.99, 850 - 854.19 code in any DX position or DX V57xx as principal DX Code | G45.0, G45.1, G45.2, G45.8, G45.9, G46.0, G46.1, G46.2, G46.3, G46.4, G46.5, G46.6, G46.7, G46.8, G97.31, G97.32, I60.00, I60.01, I60.02, I60.10, I60.11, I60.12, I60.20, I60.21, I60.22, I60.30, I60.31, I60.32, I60.4, I60.50, I60.51, I60.52, I60.6, I60.7, I60.8, I60.9, I61.0, I61.1, I61.2, I61.3, I61.4, I61.5, I61.6, I61.8, I61.9, I63.00, I63.011, I63.012, I63.013, I63.019, I63.02, I63.031, I63.032, I63.039, I63.09, I63.10, I63.111, I63.112, I63.113, I63.119, I63.12, I63.131, I63.132, I63.133, I63.139, I63.19, I63.20, I63.211, I63.212, I63.213, I63.219, I63.22, I63.231, I63.232, I63.233, I63.239, I63.29, I63.30, I63.311, I63.312, I63.313, I63.319, I63.321, I63.322, I63.323, I63.329, I63.331, I63.332, I63.333, I63.339, I63.341, I63.342, I63.343, I63.349, I63.39, I63.40, I63.411, I63.412, I63.413, I63.419, I63.421, I63.422, I63.423, I63.429, I63.431, I63.432, I63.433, I63.439, I63.441, I63.442, I63.443, I63.449, I63.49, I63.50, I63.511, I63.512, I63.513, I63.519, I63.521, I63.522, I63.523, I63.529, I63.531, I63.532, I63.533, I63.539, I63.541, I63.542, I63.543, I63.549, I63.59, I63.6, I63.8, I63.81, I63.89, I63.9, I66.01, I66.02, I66.03, I66.09, I66.11, I66.12, I66.13, I66.19, I66.21, I66.22, I66.23, I66.29, I66.3, I66.8, I66.9, I67.841, I67.848, I67.89, I97.810, I97.811, I97.820, I97.821  Exclusion criteria: any S01.90XA, S02.0XXA, S02.0XXB, S02.101A, S02.101B, S02.102A, S02.102B, S02.109A, S02.109B, S02.10XA, S02.10XB, S02.110A, S02.110B, S02.111A, S02.111B, S02.112A, S02.112B, S02.113A, S02.113B, S02.118A, S02.118B, S02.119A, S02.119B, S02.11GA, S02.11GB, S02.11HA, S02.11HB, S02.121A, S02.121B, S02.121D, S02.121G, S02.121K, S02.121S, S02.122A, S02.122B, S02.122D, S02.122G, S02.122K, S02.122S, S02.129A, S02.129B, S02.129D, S02.129G, S02.129K, S02.129S, S02.19XA, S02.19XB, S02.2XXA, S02.2XXB, S02.30XA, S02.30XB, S02.31XA, S02.31XB, S02.32XA, S02.32XB, S02.3XXA, S02.3XXB, S02.400A, S02.400B, S02.401A, S02.401B, S02.402A, S02.402B, S02.40AA, S02.40AB, S02.40BA, S02.40BB, S02.40CA, S02.40CB, S02.40DA, S02.40DB, S02.40EA, S02.40EB, S02.40FA, S02.40FB, S02.411A, S02.411B, S02.412A, S02.412B, S02.413A, S02.413B, S02.42XA, S02.42XB, S02.600A, S02.600B, S02.601A, S02.601B, S02.602A, S02.602B, S02.609A, S02.609B, S02.610A, S02.610B, S02.611A, S02.611B, S02.612A, S02.612B, S02.61XA, S02.61XB, S02.620A, S02.620B, S02.621A, S02.621B, S02.622A, S02.622B, S02.62XA, S02.62XB, S02.630A, S02.630B, S02.631A, S02.631B, S02.632A, S02.632B, S02.63XA, S02.63XB, S02.640A, S02.640B, S02.641A, S02.641B, S02.642A, S02.642B, S02.64XA, S02.64XB, S02.650A, S02.650B, S02.651A, S02.651B, S02.652A, S02.652B, S02.65XA, S02.65XB, S02.66XA, S02.66XB, S02.670A, S02.670B, S02.671A, S02.671B, S02.672A, S02.672B, S02.67XA, S02.67XB, S02.69XA, S02.69XB, S02.80XA, S02.80XB, S02.81XA, S02.81XB, S02.82XA, S02.82XB, S02.831A, S02.831B, S02.831D, S02.831G, S02.831K, S02.831S, S02.832A, S02.832B, S02.832D, S02.832G, S02.832K, S02.832S, S02.839A, S02.839B, S02.839D, S02.839G, S02.839K, S02.839S, S02.841A, S02.841B, S02.841D, S02.841G, S02.841K, S02.841S, S02.842A, S02.842B, S02.842D, S02.842G, S02.842K, S02.842S, S02.849A, S02.849B, S02.849D, S02.849G, S02.849K, S02.849S, S02.85XA, S02.85XB, S02.85XD, S02.85XG, S02.85XK, S02.85XS, S02.8XXA, S02.8XXB, S02.91XA, S02.91XB, S02.92XA, S02.92XB, S06.0X0A, S06.0X1A, S06.0X2A, S06.0X3A, S06.0X4A, S06.0X5A, S06.0X6A, S06.0X7A, S06.0X8A, S06.0X9A, S06.1X0A, S06.1X1A, S06.1X2A, S06.1X3A, S06.1X4A, S06.1X5A, S06.1X6A, S06.1X7A, S06.1X8A, S06.1X9A, S06.2X0A, S06.2X1A, S06.2X2A, S06.2X3A, S06.2X4A, S06.2X5A, S06.2X6A, S06.2X7A, S06.2X8A, S06.2X9A, S06.300A, S06.301A, S06.302A, S06.303A, S06.304A, S06.305A, S06.306A, S06.307A, S06.308A, S06.309A, S06.310A, S06.311A, S06.312A, S06.313A, S06.314A, S06.315A, S06.316A, S06.317A, S06.318A, S06.319A, S06.320A, S06.321A, S06.322A, S06.323A, S06.324A, S06.325A, S06.326A, S06.327A, S06.328A, S06.329A, S06.330A, S06.331A, S06.332A, S06.333A, S06.334A, S06.335A, S06.336A, S06.337A, S06.338A, S06.339A, S06.340A, S06.341A, S06.342A, S06.343A, S06.344A, S06.345A, S06.346A, S06.347A, S06.348A, S06.349A, S06.350A, S06.351A, S06.352A, S06.353A, S06.354A, S06.355A, S06.356A, S06.357A, S06.358A, S06.359A, S06.360A, S06.361A, S06.362A, S06.363A, S06.364A, S06.365A, S06.366A, S06.367A, S06.368A, S06.369A, S06.370A, S06.371A, S06.372A, S06.373A, S06.374A, S06.375A, S06.376A, S06.377A, S06.378A, S06.379A, S06.380A, S06.381A, S06.382A, S06.383A, S06.384A, S06.385A, S06.386A, S06.387A, S06.388A, S06.389A, S06.4X0A, S06.4X1A, S06.4X2A, S06.4X3A, S06.4X4A, S06.4X5A, S06.4X6A, S06.4X7A, S06.4X8A, S06.4X9A, S06.5X0A, S06.5X1A, S06.5X2A, S06.5X3A, S06.5X4A, S06.5X5A, S06.5X6A, S06.5X7A, S06.5X8A, S06.5X9A, S06.6X0A, S06.6X1A, S06.6X2A, S06.6X3A, S06.6X4A, S06.6X5A, S06.6X6A, S06.6X7A, S06.6X8A, S06.6X9A, S06.810A, S06.811A, S06.812A, S06.813A, S06.814A, S06.815A, S06.816A, S06.817A, S06.818A, S06.819A, S06.820A, S06.821A, S06.822A, S06.823A, S06.824A, S06.825A, S06.826A, S06.827A, S06.828A, S06.829A, S06.890A, S06.891A, S06.892A, S06.893A, S06.894A, S06.895A, S06.896A, S06.897A, S06.898A, S06.899A, S06.9X0A, S06.9X1A, S06.9X2A, S06.9X3A, S06.9X4A, S06.9X5A, S06.9X6A, S06.9X7A, S06.9X8A, S06.9X9A code in any DX position or Z51.89 as principal DX Code | >= 1 inpatient claim **OR** 2 HOP / Carrier claims with DX codes |
| **ADRD** | 3 | 331.0, 331.11, 331.19, 331.2, 331.7, 290.0, 290.10, 290.11, 290.12, 290.13, 290.20, 290.21, 290.3, 290.40, 290.41, 290.42, 290.43, 294.0, 294.10, 294.11, 294.20, 294.21, 294.8, 797 | F01.50, F01.51, F02.80, F02.81, F03.90, F03.91, F04, F05, F06.1, F06.8, G13.8, G30.0, G30.1, G30.8, G30.9, G31.01, G31.09, G31.1, G31.2, G94, R41.81, R54 | >= 1 inpatient, SNF, HHA, HOP, or Carrier claim with DX code |

Notes. Ref. period = references period, DX = diagnosis. SNF = Skilled Nursing Facility, HHA = Home Health Agency, HOP = Hospital Outpatient, ICD = International Classification of Diseases, AMI = Acute myocardial infarction, AFIB = Atrial fibrillation, RA/OA = Rheumatoid or osteoarthritis, HF = Heart failure, STIA = Stroke, or transient ischemic attack, IHD = Ischemic heart disease, HTN = Hypertension, ADRD = Alzheimer’s disease or related dementias. If not indicated otherwise, any diagnostic code on the claim within the reference period qualifies for coding. Table adapted from https://www2.ccwdata.org/web/guest/condition-categories-chronic (2/12/2024).

**Table S2.** Participants from the Health and Retirement Survey characteristics at baseline out-of-sample due to missing information in HRS covariate data or change in Medicare fee-for-service enrollment that led to censoring and exclusion from primary analyses (total n=9,134).

| **Characteristic** | **Summary Measures** | **n Total** |
| --- | --- | --- |
| **Age in years, mean (SD)** | 71.1 (5.6) | 9,134 |
| **Female, n (%)** | 5,058 (55.4) | 9,134 |
| **Race/ethnicity** |  |  |
| **Black, n (%)** | 1,361 (14.9) | 9,134 |
| **Hispanic, n (%)** | 934 (10.2) | 9,134 |
| **Twelve or more years of education, n (%)** | 6,343 (69.6) | 9,119 |
| **Coupled, n (%)** | 5,178 (64.8) | 7,997 |
| **Net worth, median (IQR)** | $134 600 ($37 500, $366 000) | 6,634 |
| **Body Mass Index, mean (SD)** | 27.6 (5.3) | 6,278 |
| **Two or more ADL limitations, n (%)** | 647 (9.8) | 6,626 |

Notes. SD = standard deviation. IQR = Interquartile range, ADL = activities of daily living.

**Table S3.** Distribution of chronic conditions at baseline of Health and Retirement Survey participants out-of-sample due to missing information in HRS covariate data, or change in Medicare fee-for-service enrollment that led to censoring and exclusion from primary analyses.

| **Characteristic** | **n (%)** | **n Total** |
| --- | --- | --- |
| **Acute myocardial infarction** | 73 (2.3%) | n=3,114 |
| **Atrial fibrillation** | 203 (6.5%) | n=3,114 |
| **Diabetes** | 665 (24.5%) | n=2,710 |
| **Heart failure** | 437 (16.1%) | n=2,710 |
| **Hypertension** | 1,895 (60.9%) | n=3,114 |
| **Ischemic heart disease** | 937 (34.6%) | n=2,710 |
| **Rheumatoid or osteoarthritis** | 842 (31.1%) | n=2,710 |
| **Stroke, or transient ischemic attack** | 210 (6.7%) | n=3,114 |
| **Number of conditions at baseline** |  |  |
| **0** | 622 (23.0%) | n=2,710 |
| **1** | 694 (25.6%) |  |
| **2** | 575 (21.2%) |  |
| **3** | 383 (14.1%) |  |
| **4** | 250 (9.2%) |  |
| **5** | 112 (4.1%) |  |
| **6+** | 74 (2.7%) |  |

**Table S4.** Summary of participants with or without conditions including risk ratios (RR), adjusted odds ratios (aOR) with 95% confidence intervals (CI) and population attributable fractions (PAF).

| **Condition** | **n exposed (with ADRD)** | **n unexposed (with ADRD)** | **RR** | **aOR (95% CI)** | **PAF_RR_** | **PAF_OR_** |
| --- | --- | --- | --- | --- | --- | --- |
| **Acute myocardial infarction** | 914 (231) | 9,030 (1,796) | 1.27 | 1.14 (0.98; 1.32) | 2.43 | 1.36 |
| **Atrial fibrillation** | 2,147 (531) | 7,797 (1,496) | 1.29 | 1.20 (1.08; 1.34) | 5.87 | 4.41 |
| **Heart failure** | 4,186 (1,058) | 5,758 (969) | 1.50 | 1.47 (1.32; 1.63) | 17.4 | 16.6 |
| **Diabetes** | 3,727 (803) | 6,217 (1,224) | 1.09 | 1.14 (1.04; 1.26) | 3.42 | 4.95 |
| **Hypertension** | 8,370 (1,817) | 1,574 (210) | 1.63 | 1.27 (1.09; 1.49) | 34.5 | 19.2 |
| **Ischemic heart disease** | 6,135 (1,449) | 3,809 (578) | 1.56 | 1.22 (1.09; 1.37) | 25.6 | 12.9 |
| **Rheumatoid or osteoarthritis** | 6,027 (1,414) | 3,917 (613) | 1.50 | 1.28 (1.15; 1.42) | 23.2 | 15.2 |
| **Stroke, or transient ischemic attack** | 2,248 (705) | 7,696 (1,322) | 1.83 | 1.96 (1.78; 2.17) | 15.7 | 17.1 |


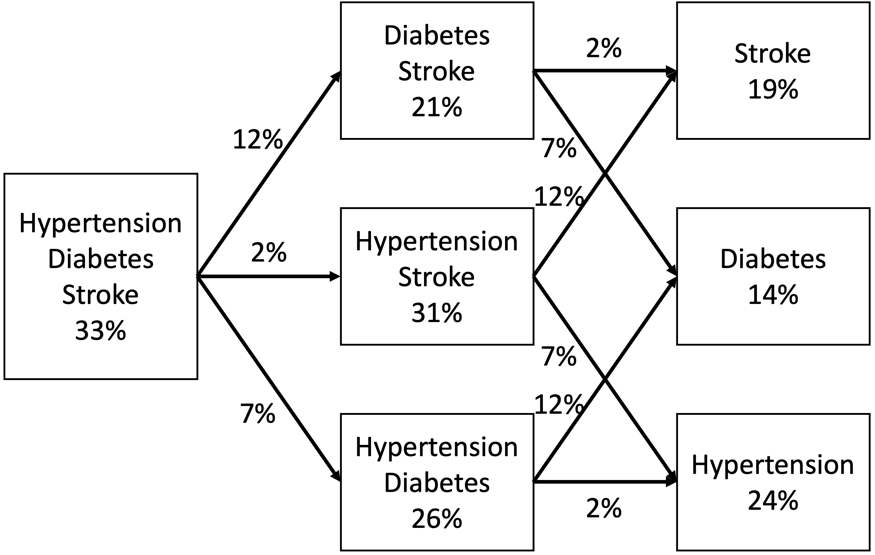


| **Removal Sequence** | | | **Sequential Attributable Fraction** | | |
| --- | --- | --- | --- | --- | --- |
| 1^st^ | 2^nd^ | 3^rd^ | Hypertension | Diabetes | Stroke |
| H | D | S | 12 | 2 | 19 |
| D | H | S | 12 | 2 | 19 |
| H | S | D | 12 | 14 | 7 |
| S | H | D | 12 | 14 | 7 |
| D | S | H | 24 | 2 | 7 |
| S | D | H | 24 | 2 | 7 |
|  |  |  |  |  |  |
| Average Attributable Fraction at time *t* | | | 16 | 6 | 11 |

**Figure S1**. Example computation of average attributable fraction at time t for a hypothetical stratum k consisting of hypertension (H), stroke (S), and diabetes (D). Equation (4) of Box S1 corresponds to the highlighted path and respective cells. Figure adapted from ^3^.


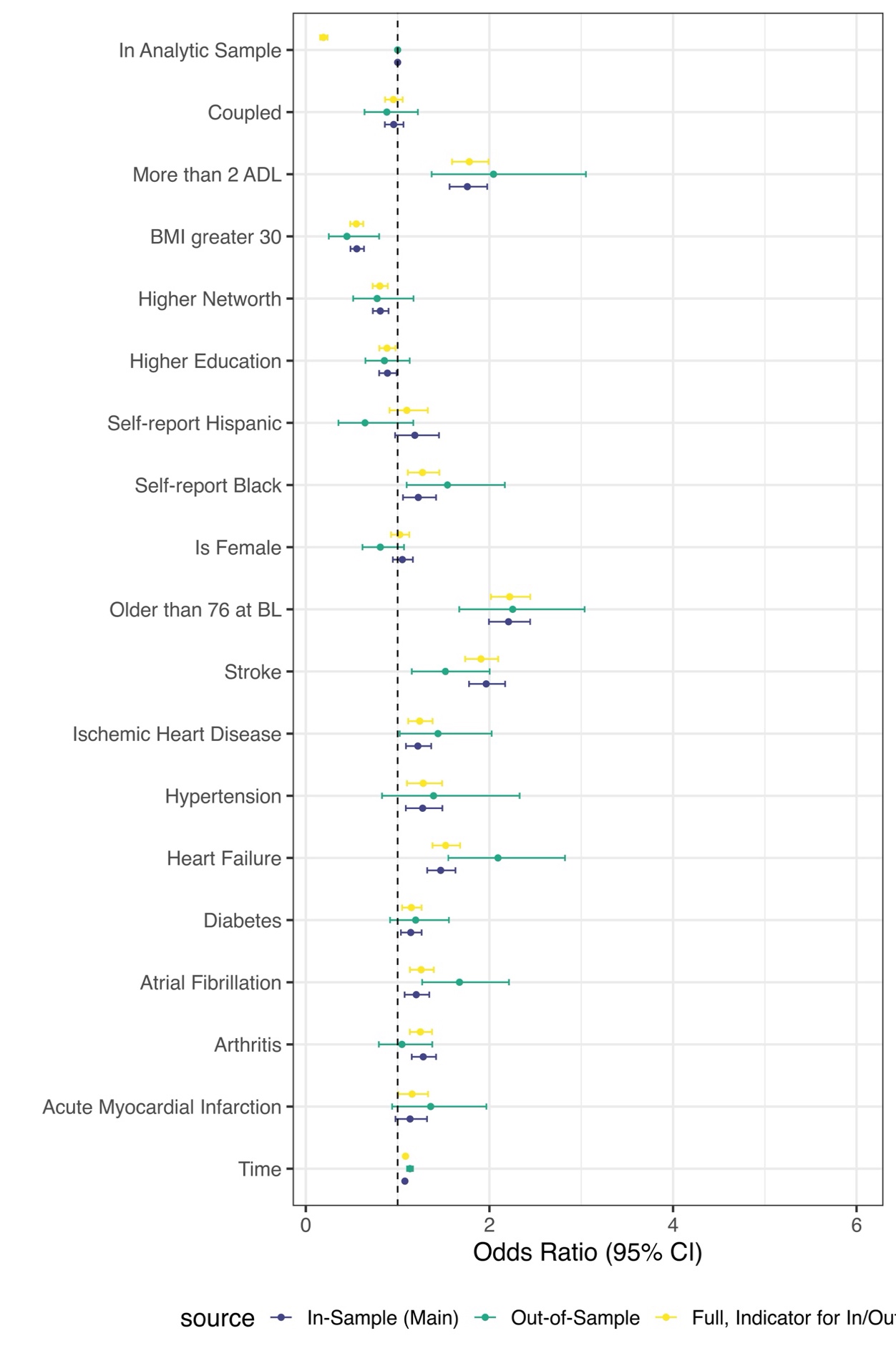

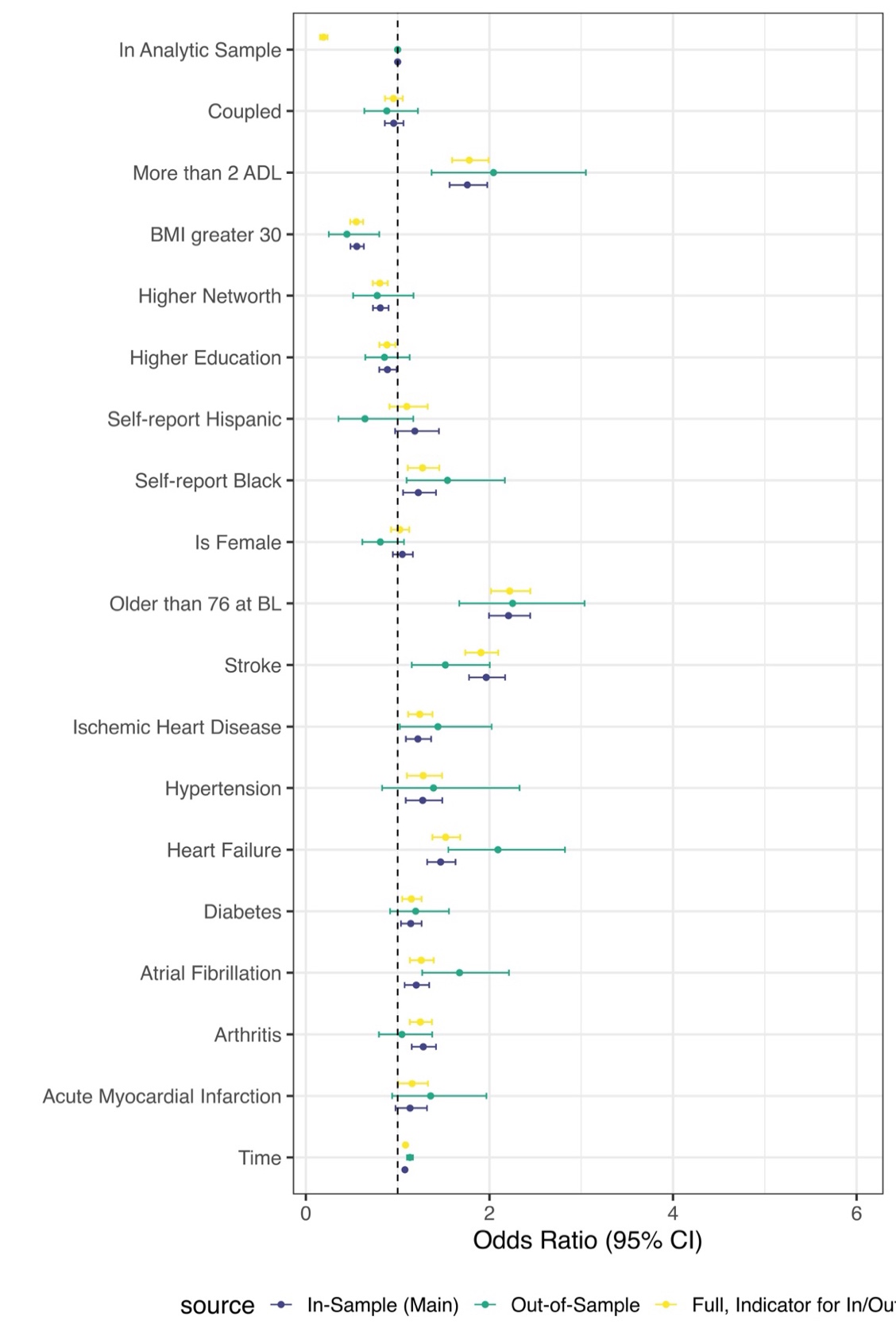
s

**Figure S2**. Odds ratios with 95% confidence intervals for pooled logistic regression models with in- (n=9,944) and out-of-sample (n=9,134) participants. In-sample estimates are based on data from participants that were included in primary analyses, out-of-sample estimates are based on data from participants that were excluded due to missing information in HRS covariate data, or change in Medicare fee-for-service enrollment that led to censoring and exclusion from primary analyses (proc logistic with missing option, estimates for missing vs. reference not depicted). Full estimates are based on data from both (n=19,078), with an additional binary variable denoting inclusion in primary analyses (In Analytic Sample). Note that the indicator (In Analytic Sample) is only estimated in the full data model (yellow).
**Abbreviations:** ADL=activities of daily living; BMI=body mass index; BL=baseline; CI=confidence intervals
